# Enhanced Therapeutic Efficacy of Combining Losartan and Chemo-Immunotherapy for Triple Negative Breast Cancer

**DOI:** 10.3389/fimmu.2022.938439

**Published:** 2022-06-23

**Authors:** Qing Zhao, Xuexin He, Xiyi Qin, Yu Liu, Han Jiang, Jing Wang, Shuang Wu, Rui Zhou, Congcong Yu, Suling Liu, Hong Zhang, Mei Tian

**Affiliations:** ^1^Department of Nuclear Medicine and Positron Emission Tomography (PET) Center, The Second Affiliated Hospital of Zhejiang University School of Medicine, Hangzhou, China; ^2^Institute of Nuclear Medicine and Molecular Imaging of Zhejiang University, Hangzhou, China; ^3^Key Laboratory of Medical Molecular Imaging of Zhejiang Province, Hangzhou, China; ^4^Department of Medical Oncology, Huashan Hospital of Fudan University, Shanghai, China; ^5^Positron Emission Tomography-Computed Tomography (PET-CT) Center, Fujian Medical University Union Hospital, Fuzhou, China; ^6^Fudan University Shanghai Cancer Center & Institutes of Biomedical Sciences; State Key Laboratory of Genetic Engineering; Cancer Institutes; Fudan University, Shanghai, China; ^7^College of Biomedical Engineering & Instrument Science, Zhejiang University, Hangzhou, China; ^8^Key Laboratory for Biomedical Engineering of Ministry of Education, Zhejiang University, Hangzhou, China; ^9^Human Phenome Institute, Fudan University, Shanghai, China

**Keywords:** triple-negative breast cancer, immunotherapy, extracellular matrix, chemotherapy, positron emission tomography (PET)

## Abstract

Triple-negative breast cancer (TNBC) is a particularly aggressive subtype of breast cancer, which is relatively resistant to anti-programmed cell death-1 (α-PD1) therapy, characterized as non-immunogenic, dense stroma and accumulation of M2 tumor-associated macrophages (TAMs). Despite progress in strategies to deplete extracellular matrix (ECM) and enhance tumor-cell immunogenicity, the combinatorial anti-cancer effects with α-PD1 need to be explored. Here, we applied doxorubicin hydrochloride liposome (Dox-L) as immunogenic cell death (ICD)-inducing nano-chemotherapy and used losartan as stroma-depleting agent to improve α-PD1 efficacy (Losartan + Dox-L + α-PD1). The results showed that losartan could cause ECM reduction, facilitating enhanced delivery of Dox-L and further dendritic cell (DC) maturation. Additionally, losartan could also alleviate hypoxia for TNBC, thus reprogramming pro-cancer M2 TAMs to anti-cancer M1 TAMs, successfully overcoming immune-suppressive microenvironment. These modifications led to a significant increase in T cells’ infiltration and augmented anti-tumor immunity as exemplified by the notable reduction in tumor size and lung metastases. In summary, our findings support that combined treatment of losartan with Dox-L normalizes immunological-cold microenvironment, improves immuno-stimulation and optimizes the efficacy of TNBC immunotherapy. A novel combinational strategy with FDA-approved compounds proposed by the study may potentially be useful in TNBC clinical treatment.

## Introduction

Immune checkpoint blockade (ICB), such as anti-programmed cell death-1 (α-PD1) therapy, has achieved remarkable success to fight cancer in the clinic (1, 2), with moderate responses occurring in immunogenic (hot) tumor model (3–5). However, the non-immunogenic (cold) tumors, e.g., triple-negative breast cancer (TNBC), are relatively resistant to ICB therapy (6, 7). TNBC is generally regarded as a particularly aggressive subtype of breast cancer with rapid progression of disease, early attack of metastasis and unfavorable survival outcomes (8, 9). Therefore, the development of strategies to enhance the efficacy of ICB in TNBC is crucial and urgent (7, 10). The immune-suppressive TNBC microenvironment lies on the fact that tumor cell itself is absent of immunogenic nature (11, 12), thereby failing to activate anti-cancer immunity (13). It has been reported that certain chemotherapeutic agents induce immunogenic cell death (ICD) of tumor (14–16). ICD provides stimuli to facilitate tumor antigen cross-presentation for dendritic cell (DC) maturation, which possesses the ability to present tumor antigens to naive T cell (17, 18). Doxorubicin hydrochloride liposome (Dox-L) is approved by the Food and Drug Administration (FDA) and applied in multiple clinical trials as ICD-inducing nanomedicine (19, 20). Nevertheless, the penetration, intra-tumoral distribution and therapeutic outcome of Dox-L were hindered by the dense tumor stroma (16, 21), leading to compromised tumor-killing capacity and ICD effect. Thus, the strategy for stroma normalization is needed to increase anti-tumor activity. The dense tumor extracellular matrix (ECM) acts as major barrier for effective drug delivery. Additionally, the ECM plays a pivotal role in the immune-suppressive tumor microenvironment (22, 23). The hypo-perfusion arising from intra-tumoral mechanical forces causing vessel compression results in the reduction of the number of immune cells infiltrating into the tumor (24, 25). Also, inadequate oxygen supply caused by the impaired tumor blood vessels results in tumor hypoxia and hypoxia-mediated immune exhaustion, attenuating the killing ability of effector immune cells (26, 27). Hypoxic regions of solid tumors could promote the polarization of tumor-promoting (M2-like) phenotype tumor-associated macrophages (TAMs) from pro-inflammatory, antitumor (M1-like) phenotype TAMs (28–30). Previous studies suggested that losartan, an inexpensive antihypertensive and antifibrotic common drug, could potentially be used to improve the efficacy of various nanotherapeutics in multiple tumor types. Losartan had the potential to lessen solid stress, decompress the intratumor vessels, enhance oxygen and drug delivery and raise chemotherapy efficacy for solid tumors (21, 31, 32).

To date, little evidence has shown that combinatorial use of losartan and Dox-L could augment ICB-mediated immunotherapy against TNBC. The purpose of our study was to explore whether the normalization effects of losartan and the ICD effect of Dox-L can be optimized with ICB therapy. We demonstrate that combination of losartan, Dox-L and α-PD1 therapy reprogrammed tumor microenvironment from non-immunogenic to immunogenic. Finally, by depleting tumor stroma and overcoming hypoxia, the combined strategy produces ICD effect, improves T cell recruitment, and suppresses the formation of M2 phenotype TAMs, thus boosting the anti-cancer effect significantly.

## Material and Methods

### Cell Culture

The 4T1 and EMT6 murine TNBC cell lines were obtained from the Cell Bank of Chinese Academy of Science (Shanghai, China). Both cell lines were cultured in RPMI 1640 medium supplemented with 10% fetal bovine serum (FBS) and 1% antibiotics and were incubated at 37°C with 5% CO_2_ atmosphere.

### Mice and Tumor Models

This study complied with all ethical animal testing and research regulations, with study guidelines reviewed and approved by the Laboratory Animal Center of Zhejiang University (NO. ZJU20210070 and NO. ZJU20210232) and was in line with the regulations of the National Ministry of Health. BALB/c mice (6~8 weeks old, female) were purchased from Ziyuan Laboratory Animal in Hangzhou, China. 4T1 cells or EMT6 cells (5 × 10^5^) suspended in 50 μL of PBS were subcutaneously injected into the right mammary fat pad.

### *In Vitro* Experiments

#### *In Vitro* Cytotoxicity

4T1 cells or EMT6 cells were seeded in 96-well plates at a density of 3 × 10^3^ cells per well and allowed to adhere for 24h. Cells were then treated with different concentrations of losartan (Absin, China) and Dox-L (Shanghai Fudan-Zhangjiang Bio-Pharmaceutical Co., Ltd., China). Cell viability was measured by MTT Cell Proliferation and Cytotoxicity Assay Kit (Beyotime, China) according to the absorbance at the wavelength of 570 nm with a microplate reader (Bio-Rad, USA).

#### *In Vitro* CRT Exposure Analysis

To assess CRT expression by flow cytometry, 4T1 cells or EMT6 cells inoculated in six-well plates (5 × 10^5^ cells/well) were cultured with different concentrations of losartan and Dox-L for 48h. The treated cells were collected and sequentially incubated with a primary rabbit Alexa Fluor^®^ 647-conjugated anti-CRT antibody (dilution 1:50, Abcam, UK) for 20 min at room temperature. The cells were incubated in 500µL PBS containing 10% fetal bovine serum (FBS) and 1µg/mL DAPI (Biolegend, USA) before assessment with a flow cytometer (CytoFLEX LX, Beckman Coulter, USA). The mean fluorescence intensity of stained cells was gated on DAPI- cells.

#### Detection of HMGB1 Release

4T1 cells or EMT6 cells inoculated in six-well plates (3 × 10^5^ cells/well) were cultured with losartan at a dose of 100μg/ml and Dox-L at a dose of 200μg/ml for 48h. The cell culture supernatant was harvested for quantification of HMGB1 by Mouse/Rat HMGB1 ELISA Kit based on the manufacturer’s instructions (Arigo, China).

### *In Vivo* Biodistribution Analysis

For *in vivo* Dox-L biodistribution analysis, 4T1 tumor-bearing mice were randomly distributed into two groups (n = 3) for different treatment: Dox-L (control) and Losartan + Dox-L. The 4T1 tumor-bearing mice from the group Losartan + Dox-L were injected intraperitoneally with 200µL 4 mg/mL losartan for 5 days before Dox-L injection. The 4T1 tumor-bearing mice from both groups received injection with Dox-L at a dose of 5mg/kg *via* the tail vein. Tumors were surgically excised at 12h post-injection to make frozen sections. Staining for blood vessels was done by incubation with rabbit anti-CD31 mouse monoclonal antibody (mAb) (dilution 1:200, Cell Signaling Technology, USA) and Alexa Fluor^®^647-conjugated anti-rabbit secondary antibody (dilution 1:500, Cell Signaling Technology, USA). DAPI was applied to stain cell nuclei (dilution 1:5000, USA). The images were captured with confocal microscopy (Nikon A1 Ti, Japan).

### *In Vivo* Tumor Growth Inhibition and Lung Metastasis Suppression

#### Treatment Protocols

For *in vivo* chemotherapy, 4T1 tumor-bearing mice were randomly distributed into four groups (n = 5) for injection of various agents: (1) Saline (control), (2) Losartan (40mg/kg, i.p., daily for 8 days), (3) Dox-L (5mg/kg, i.v., once every 3 days for up to 3 doses), (4) Losartan + Dox-L. The losartan powder was dissolved in saline to obtain a concentration of 4 mg/mL, and the injection volume was 200µL. The Dox-L was diluted in saline to obtain a concentration of 1mg/mL, and the injection volume was 100µL.

For *in vivo* chemo-immunotherapy, 4T1 or EMT6 tumor-bearing mice were randomly distributed into six groups (n = 5) for injection of various agents: (1) Saline (control), (2) α-PD1 (10mg/kg, i.p., once every 3 days for up to 3 doses, BioXCell, USA), (3) Losartan + α-PD1, (4) Losartan + Dox-L, (5) Dox-L + α-PD1, (6) Losartan + Dox-L + α-PD1. The injection for losartan and Dox-L was conducted following the same procedure as described above. The α-PD1 was diluted in saline to obtain a concentration of 2mg/mL, and the injection volume was 100µL.

#### Antitumor Vaccination

To study the immune memory effect, the BALB/c mice (n = 3) treated with losartan combined chemo-immunotherapy were rechallenged with 5 × 10^5^ 4T1 cells on the left mammary fat pad 1 month after the tumors disappeared. Meanwhile, the 4T1 orthotopic murine breast cancer model was also established in three BALB/c mice as the control group.

#### *In Vivo* Antitumor Efficacy

The tumor growth and body weights were monitored every 3 days for 17 days. Tumor size was measured with a digital caliper, and tumor volumes were calculated by the following formula: volume = (width^2^ × length)/2. The ^18^F-FDG PET/CT images of the mice were collected on day -1, day 7, and day 17 to investigate the anti-tumor effects of chemotherapy. To study the lung metastasis, the lungs were harvested and were fixed in Bouin’s solution. The metastatic lung tumors were directly counted through microscopic observation and subsequently studied by pathological histologic evaluation. The major organ tissue slices were stained by H&E following the standard protocol. Prior to organ excision and sacrifice, the mice were anesthetized with Avertin (200mg/kg, i.p.).

### *In Vivo* Immunohistochemical Assessment of Tumor Microenvironment

#### Tumor Collection and Embedding

The 4T1 tumor-bearing mice were treated with different strategies at the abovementioned doses for up to one dose of α-PD1 and/or Dox-L. The tumors for immunofluorescence staining analysis and quantification were collected from mice, fixed in 4% paraformaldehyde for 24h, whereafter soaked in 30% sucrose solution for 24h. The tumors were embedded in optimum cutting temperature compound (OCT) (Sakura Tissue-Tek, USA) and frozen with liquid nitrogen.

#### Immunofluorescence Staining

The frozen tumors were cut into 10μm sections for immunofluorescence staining and confocal imaging. Staining for blood vessels was achieved by incubation with anti-CD31 mouse mAb conjugated to Alexa Fluor^®^488 (dilution 1:200, Santa Cruz, USA). Anti-smooth muscle actin mouse mAb conjugated to Alexa Fluor^®^647 (dilution 1:200, Santa Cruz, USA) was used for detection of α-SMA. Collagen I was detected with the anti-COL1A1 mouse mAb (dilution 1:200, Santa Cruz, USA) and Alexa Fluor^®^488-conjugated goat anti-mouse secondary antibody (dilution 1:500, Absin, China). HIF-1α was detected with the anti-HIF-1α rabbit mAb (dilution 1:200, Cell Signaling Technology, USA) and Alexa Fluor^®^488-conjugated goat anti-rabbit secondary antibody (dilution 1:500, Cell Signaling Technology, USA). PD1 was detected with the anti-PD1 rabbit mAb (dilution 1:200, Cell Signaling Technology, USA) and Alexa Fluor^®^647-conjugated goat anti-rabbit secondary antibody (dilution 1:500, Cell Signaling Technology, USA). Cell nuclei were marked with DAPI staining (dilution 1:5000, Biolegend, USA). The images were captured at 20× magnification *via* confocal microscopic examination. The intensity values of the aim indicators were quantified with Image J software.

### *In Vivo* Immune Response Analysis

#### Immunofluorescence Staining

Staining for CD8 was done by incubation with the anti-CD8-α mouse mAb (dilution 1:200, Santa Cruz, USA) and Alexa Fluor^®^488-conjugated goat anti-mouse secondary antibody (dilution 1:500, Absin, China). Anti-B7-2 mouse mAb conjugated to Alexa Fluor^®^488 (dilution 1:200, Santa Cruz, USA) was used to detect CD86. Anti-CD206 mouse mAb conjugated to Alexa Fluor^®^647 (dilution 1:200, Santa Cruz, USA) was used to detect CD206. Cell nuclei were stained with DAPI (dilution 1:5000, Biolegend, USA). The images were obtained with confocal microscopy.

#### ^18^F-FDG PET/CT Imaging and Data Analysis

The tumor-bearing mice were treated with (1) Saline, (2) α-PD1, (3) Losartan + α-PD1, (4) Losartan + Dox-L, (5) Dox-L + α-PD1, (6) Losartan + Dox-L + α-PD1 at the abovementioned doses for up to one dose of α-PD1 and/or Dox-L. The mice were kept fasting overnight, and ^18^F-FDG PET scanning was performed using a Vista eXplore (Sedecal, Spain) animal PET/CT camera system. The ^18^F-FDG probe (Calibrated doses of 12.5MBq within 100-200 μL sterile saline) was injected *via* the tail vein. Static PET scanning was performed 45 min after the probe injection. Mice were anaesthetized under 1.5% isoflurane anesthesia (mixed with 100% oxygen). The mice were placed within their cages over a temperature-controlled heating pad during the tracer uptake time and awakening time. Regions-of-interest (ROIs) were carefully delineated of the spleen as the target organ and analyzed with PMOD v4.0 software (PMOD Technologies Ltd, Zurich, Switzerland). The percentage of injected dose per gram (%ID/g) of the spleen was calculated for each ROI based on the mean ^18^F-FDG uptake. For semiquantitative analysis, ROIs were delineated on the muscle as a nontarget reference. The ratio of the mean spleen uptake to mean muscle uptake was calculated and compared between different interventions. The researcher who analyzed the data was not aware of the experimental groups of mice.

#### Flow Cytometry Assay

The mice were euthanized 24 hours after the last PET imaging timepoint. To study the immune cells in tumors, tumors from different groups were collected by surgery, then homogenized into single-cell suspensions according to the well-established procedure. To analyze the effector T cells (CD45+, CD3+, CD8+), tumor cells were stained with anti-CD45-FITC (Biolegend, USA), anti-CD3-PE (Biolegend, USA) and anti-CD8a-APC (Biolegend, USA) antibodies according to the standard protocol. TAM cells were classified into M_1_ TAM (CD11b+, F4/80+, CD86+) and M_2_ TAM cells (CD11b+, F4/80+, CD206+). For analysis of TAM cells, tumor cells were stained with anti-CD11b-PE (Biolegend, USA), anti-F4/80-FITC (Biolegend, USA), anti-CD86-Percp/Cyanine5.5 (Biolegend, USA), anti-CD206-APC (Biolegend, USA) antibodies and examined using flow cytometry. For DCs maturation (CD11c+, CD80+, CD86+) analysis, spleens collected from mice after different treatments were homogenized into single-cell suspensions and stained with anti-CD11c-FITC (Biolegend, USA), anti-CD80-APC (Biolegend, USA), anti-CD86-Percp/Cyanine5.5 antibodies for flow cytometry examination. All these antibodies used in *ex vivo* flow cytometry experiments were diluted 150 times.

#### ELISA Assay

Serum samples were harvested from mice after various treatments and diluted for ELISA analysis. The proinflammatory cytokines secreted within the blood serum, including TNF-α, IFN-γ and IL-6, were determined using ELISA kits (Biolegend, USA) according to the vendor’s instructions.

### Statistics

Data were given as means ± SEM. The statistical significance was performed by one-way analysis of variance (ANOVA) test and two-sided unpaired Student’s t-test. Correlation coefficients between acquired PET imaging parameters and the percentage of mature DC cells obtained from flow cytometry were calculated. Statistical significance was set as follows: *p < 0.05, ** *p* < 0.01, *** *p* < 0.001 and **** *p* < 0.0001. The statistical analyses were performed using GraphPad Prism (GraphPad Software v8, CA, USA).

## Results

### Dox-L Induces Anti-Tumor Effect and ICD *In Vitro*

Dox-L appeared markedly more cytotoxic than losartan in 4T1 cells (**Figures S1A, B**) and EMT6 cells (**Figures S1C, D**). The quantitative results indicate that the antitumor effect induced by Dox-L plays an important role in combination therapy. In addition, we measured levels of calreticulin (CRT) exposure and high mobility group box 1 (HMGB1) release on the 4T1 and EMT6 cells treated by different formulations.

Dox-L, but not losartan or PBS, promoted the translocation of CRT to the cell surface. Surface CRT-positive 4T1 tumor cells induced by Dox-L treatment with 100μg/mL (46.90 ± 3.04%) and 200μg/mL (51.67 ± 5.93%) concentrations were significantly more efficient than PBS treatment (17.37 ± 1.07% CRT-positive cells) (**Figures S1E, F**). Similar results were observed in EMT6 tumor cells (**Figures S1E, G**). The HMGB1 release data further confirmed the ICD induction property of Dox-L in both TNBC models (**Figures S1H, I**).

### Losartan Improves the Antitumor Efficacy of Dox-L Chemotherapy

**Figure 1:**
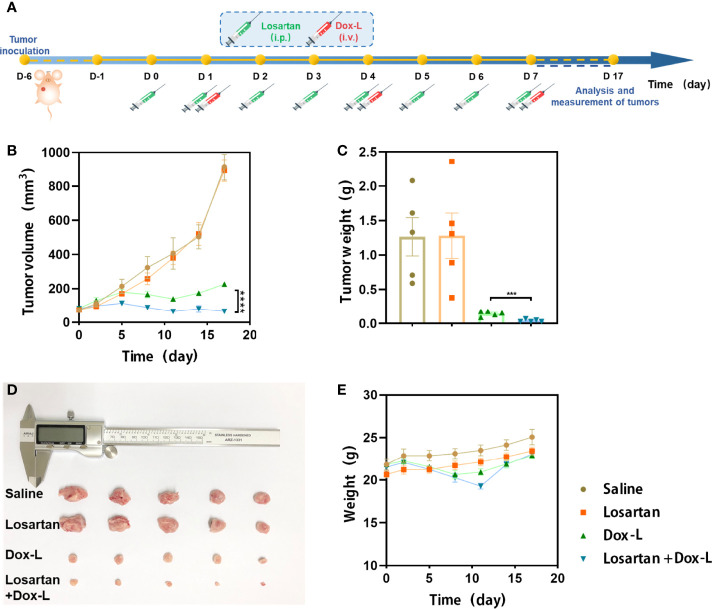
Losartan significantly inhibits the growth of tumors treated with Dox-L. **(A)** Schematic illustration of losartan and Dox-L combination therapy to tumor models (n = 5). **(B)** Average tumor growth curves of 4T1-tumor-bearing mice from different groups of mice. **(C)** Average weights of tumors at the end of treatments. **(D)** Photographs of excised tumors at the end of treatments. **(E)** Weight of mice after different treatments. Data are expressed as the mean ± SEM. Data are expressed as the mean ± SEM. Statistical significances were calculated *via* Student’s t-test, ***p < 0.001 and ****p < 0.0001.

Losartan was administered in combination with Dox-L chemotherapy to test this hypothesis as a first step. 4T1 tumor models were respectively treated with Saline (control), Losartan, Dox-L or Losartan + Dox-L. The experimental procedure, shown in **Figure 1A**. We found that losartan did not cause any evident delay in tumor growth compared to the control group. However, assessment of tumor size on the last day showed a significant reduction in tumor volume in the mice receiving the combination treatment of losartan with Dox-L compared with Dox-L monotherapy (65.30 ± 11.28mm^3^ *vs.* 226.48 ± 13.22mm^3^, *p* < 0.0001). The tumor volume trend corresponded with that of tumor weight (0.04 ± 0.01g *vs.* 0.15 ± 0.02g, *p* = 0.0005). These data demonstrate that the effect of losartan is essential to improve Dox-L anti-tumor ability (**Figures 1B–D**). Additionally, no significantly unnatural weight changes were observed in mice after Losartan + Dox-L treatment (**Figure 1E**), indicating the favorable therapeutic biosafety of the combined losartan and Dox-L.

### Combination Therapy Involved α-PD1 Immunotherapy Alleviates Primary Tumor Burden and Lung Metastases

**Figure 2:**
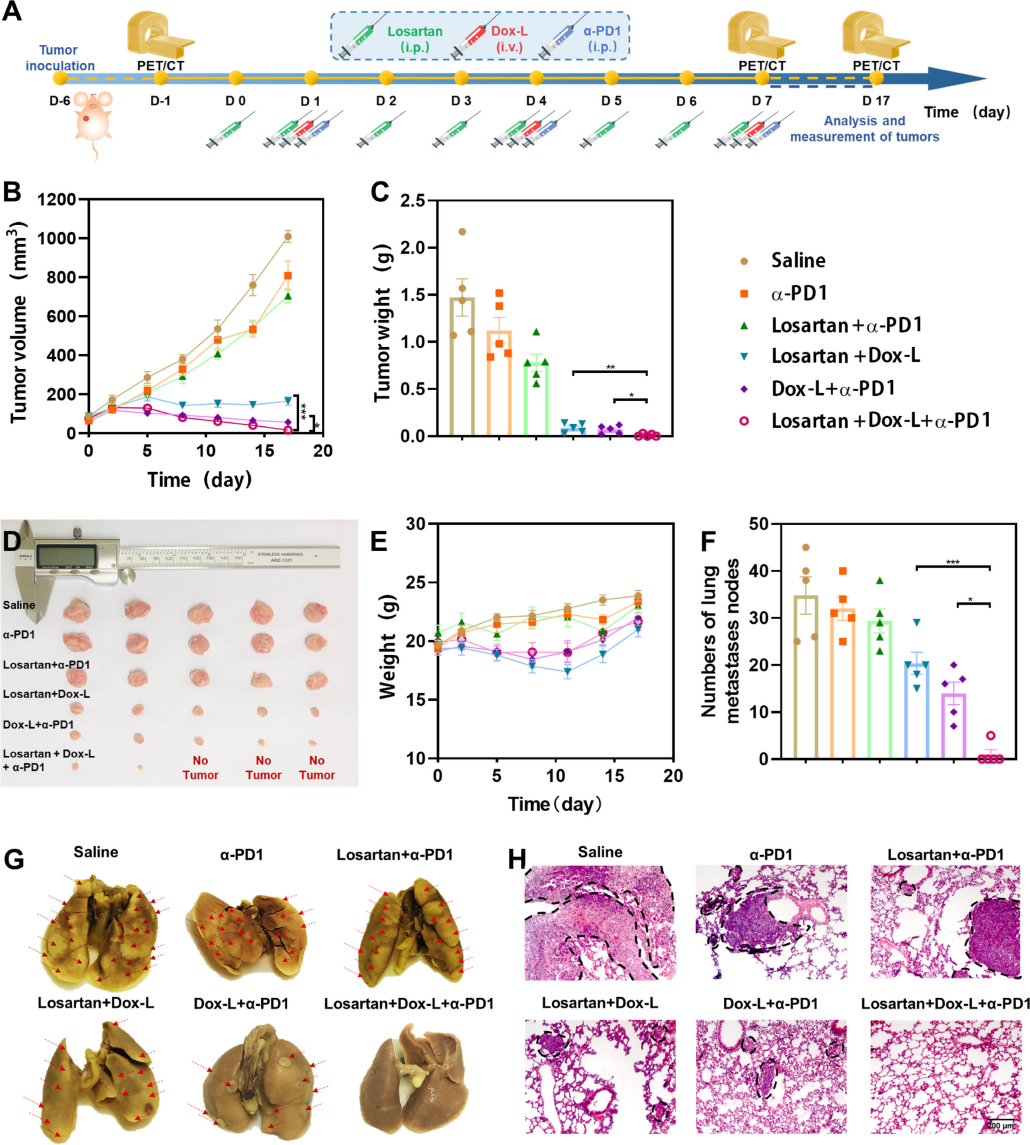
Antitumor effect of losartan, Dox-L plus anti-PD1 immunotherapy in orthotopic 4T1 tumor models. **(A)** Schematic illustration of combined treatment to tumors. (n = 5) **(B)** Tumor growth curves of different groups of orthotopic tumor-bearing mice after various treatments as indicated in the figure. **(C)** Average weights of tumors at the end of treatments. **(D)** Photographs of excised tumors at the end of treatments. **(E)** Weight of mice after different treatments. **(F)** The numbers of lung nodules were counted under an anatomy microscope (n = 5). **(G)** Representative Lung photographs of 4T1 murine breast tumors treated as indicated. Red arrows indicate the metastatic nodules on the lungs. **(H)** Representative H&E histopathological images of the lung metastasis. Scale bar = 200μm. Data are expressed as the mean ± SEM. Statistical significances were calculated *via* Student’s t-test and one-way analysis of variance (ANOVA), *p < 0.05, **p < 0.01 and ***p < 0.001.

To assess the synergistic efficiency of the combination therapy, losartan, Dox-L and α-PD1 blockade, 4T1 and EMT6 orthotopic mammary tumor models were employed. Mice bearing tumors were distributed into six groups (1) Saline (control), (2) α-PD1 (3) Losartan + α-PD1, (4) Losartan + Dox-L, (5) Dox-L + α-PD1, (6) Losartan + Dox-L + α-PD1, the treatment schedule is described in **Figure 2A**. Compared with the other groups, the volumes and weights of 4T1 tumors were significantly decreased in the Losartan + Dox-L + α-PD1 group in 4T1 tumor models (**Figures 2B–D**). The 2-[^18^F] fluoro-2-deoxy-D-glucose positron emission tomography/computed tomography (^18^F-FDG PET/CT) imaging was performed to track the growing process of the 4T1 tumor at different time points (**Figure S2**). Notably, we found three out of five mice exhibiting negligible tumor signals after the triple-combined treatment, indicating substantially inhibited tumorigenesis. Similar results were found in the EMT6 cancer model (**Figures S3A–C**). Meanwhile, there were virtually no abnormal weight changes observed in mice after Losartan + Dox-L + α-PD1 combination treatment in both 4T1 (**Figure 2E**) and EMT6 (**Figure S3D**) cancer models, indicating the high biosafety of the combined therapeutic strategy.

In addition, we examined the ability of losartan combined with chemo-immunotherapy to reduce lung metastasis in mice bearing 4T1 and EMT6 breast tumors. Macroscopic counting of metastatic lung nodules, as well as tissue section by Hematoxylin and Eosin (H&E) staining, revealed that compared to all other treatment groups, Losartan + Dox-L + α-PD1 treatment was effective in mitigating metastases (**Figures 2F–H**; **Figures S3E, F**).

Subsequently, so as to confirm the biosafety of the combinational strategy, we evaluated the probable harmful influence on normal organs caused by the combined treatment. The serum biochemistry assay was conducted, and H&E staining assay was performed on the major organs of tumor-bearing mice receiving different treatments. There was no significant difference found in serum parameters and no obvious damage found in the hearts, spleens, kidneys and livers, indicating the biosafety of the Losartan + Dox-L + α-PD1 triple therapy (**Figure S4**).

### Losartan Depletes Intra-Tumoral Dense Stroma and Hypoxia Microenvironment

**Figure 3:**
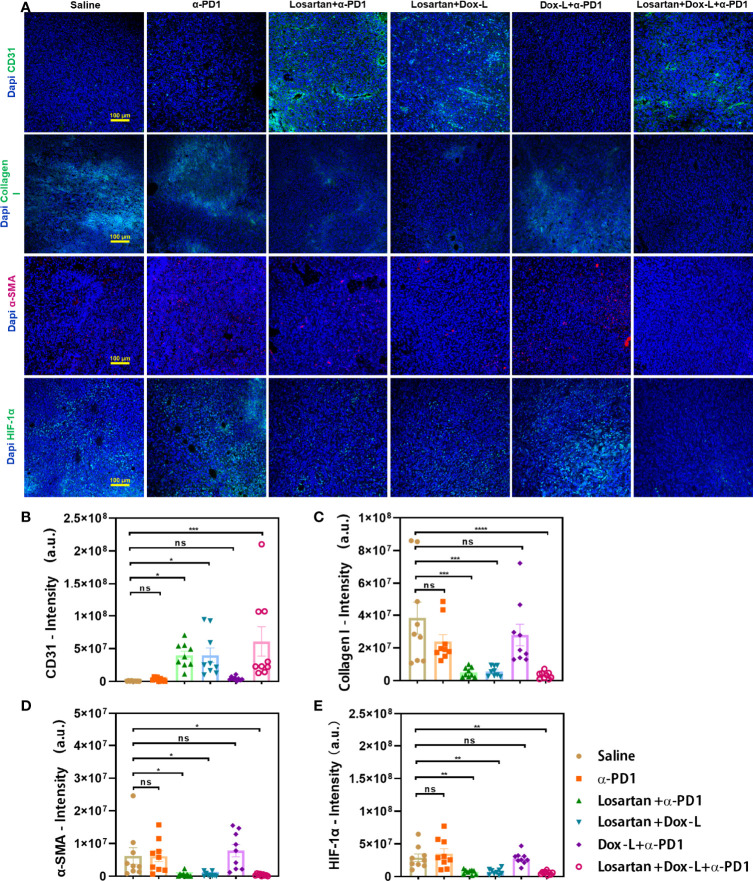
Enhanced tumor normalization by losartan relieves dense tumor stroma, increasing perfusion and alleviating tumor hypoxia. **(A)** Representative images of CD31 (endothelial marker, green), Collagen I (stroma content, green), α-SMA (pericyte marker, red), HIF-1α (hypoxia marker, green) immunofluorescence staining and DAPI (blue) nuclear staining after various treatments as indicated. Quantification of CD31 **(B)**, Collagen I **(C)**, α-SMA **(D)**, HIF-1α **(E)** positive staining signals from the images shown in **(A)**. Scale bar = 100μm. Data are expressed as the mean ± SEM. Statistical significances were calculated *via* one-way ANOVA, *p < 0.05, **p < 0.01, ***p < 0.001 (n = 9). ****p < 0.0001. ns, no significance.

To investigate if the significant antitumor effect for the Losartan + Dox-L + α-PD1 group was induced with efficient degradation of the ECM, immunofluorescence staining of tumor cryosections was performed by quantifying fluorescence intensity of main ECM components. We observed that the losartan-treated groups could increase the vascular endothelium marker CD31 and decrease the collagen and smooth muscle actin (α-SMA) content of 4T1 tumor stroma when compared with the other groups (**Figures 3A–D**). Consequently, exposed to losartan, dense tumor stroma was alleviated, and vascular perfusion was enhanced, leading to reduced tumor hypoxic microenvironment (**Figures 3A, E**). In addition, by modulating ECM, losartan significantly improved drug delivery to the tumor site of Dox-L (*p* = 0.001) (**Figure S5**).

### Combination Therapy Enhances Antitumor Immune Response

**Figure 4:**
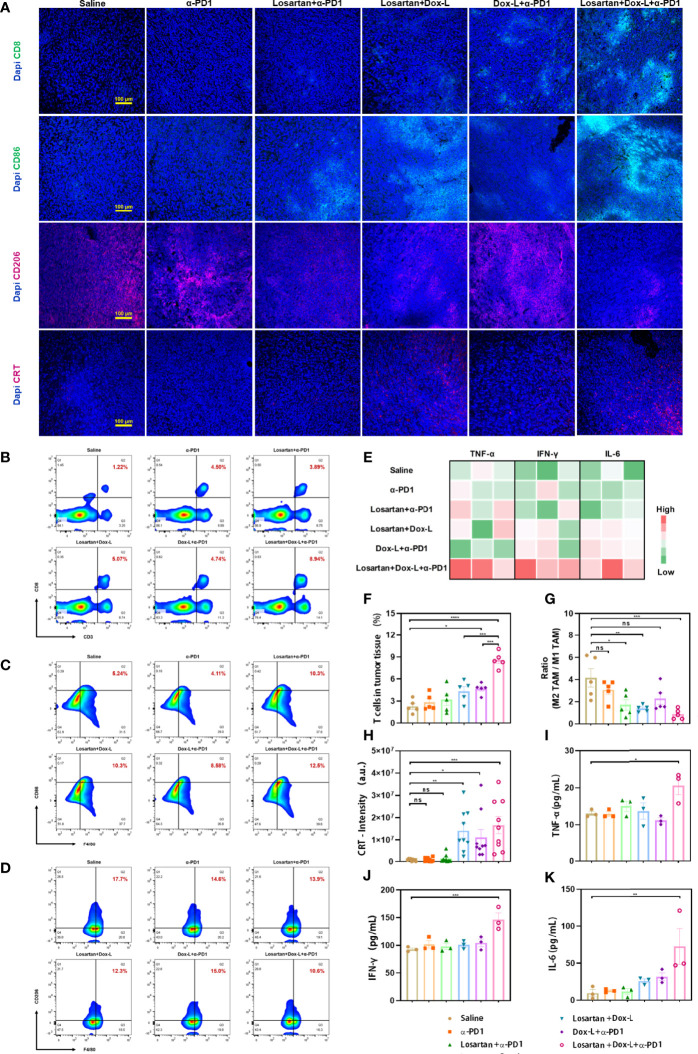
Losartan combined with chemo-immunotherapy promotes immune-stimulation in orthotopic 4T1 breast tumors. **(A)** Representative fluorescence images of 4T1 tumor slices immunostained for (effector T cell marker CD8, green), CD86 (M1-like TAM marker, green), CD206 (M2-like TAM marker, red), CRT (ICD effect marker, red) and DAPI (cell nuclei, blue). **(B–D)** Representative flow cytometry plots showing the tumor immune cells, including CD8+ T cells (CD45+, CD3+, CD8+), M1-like TAMs (CD11b+, F4/80+, CD86+) and M2-like TAMs (CD11b+, F4/80+, CD206+) in tumors after different treatments. **(E)** Heat map plot of ELISA results. **(F)** Quantification of the level of CD8+ T cells by flow cytometry analysis (n = 5). **(G)** Ratio of M2-like TAM to M1-like TAM by flow cytometry analysis (n = 5). **(H)** Quantification of CRT positive staining signals from the images shown in **(A)** (n = 9). Cytokine quantification of the secretion of TNF-α **(I)**, IFN-γ **(J)**, IL-6 **(K)** in sera from mice after various treatments as indicated in the figure (n = 3). Scale bar = 100μm. Data are expressed as the mean ± SEM. Statistical significances were calculated *via* one-way ANOVA, *p < 0.05, **p < 0.01, ***p < 0.001 and ****p < 0.0001. ns, no significance.

The antitumor immunity elicited by losartan combined with chemo-immunotherapy was further verified by the immunofluorescence assay and flow cytometry for effector T cells. It has been observed that combinational treatment instigated CD8+ T cells infiltration into 4T1 tumors effectively by immunofluorescence staining with antibodies against CD8 (**Figure 4A**). Furthermore, the quantitative changes in effector T cells (CD45+, CD3+, CD8+) were measured by flow cytometry in 4T1 and EMT6 tumor models. In both 4T1 and EMT6 models, effector T cells data showed that the Losartan + Dox-L + α-PD1 treatment led to an obvious increase compared with the other groups, 3.8-fold (8.59 ± 0.48% *vs.* 2.28 ± 0.40%, *p* < 0.0001) (**Figures 4B, F**) and 2.8-fold (7.12 ± 0.57% *vs.* 2.56 ± 0.45%, *p* < 0.0001) (**Figures S6A, E**) more effector T cells than the Saline group.

Considering the favorable efficacy of combination therapy on facilitating oxygen delivery in ECM, whether reprogramming the TAMs polarization could modulate the immune-suppressive environment in 4T1 and EMT6 tumors was investigated. Losartan-treated groups increased the anti-cancer M1-like TAMs and decreased the immune-suppressive M2-like TAM. Subsequently, the reduction of the ratio of M2-like TAMs to M1-like TAMs in losartan-treated groups was identified by flow cytometry and compared with the other groups. The M2-like TAM population was considered according to the expression of CD11b+, F4/80+, CD206+, while the M1-like TAM was according to the expression of CD11b+, F4/80+, CD86+. In 4T1 models, the values of M2/M1-like TAMs in the Losartan + α-PD1 group (1.71 ± 0.48, *p* = 0.0173), Losartan + Dox-L group (1.38 ± 0.13, *p* = 0.0054) and Losartan + Dox-L + α-PD1 group (0.85 ± 0.20, *p* = 0.0008) were significantly lower than in the saline group (4.16 ± 0.83, **Figures 4C, D, G**). Meanwhile, a comparable trend was observed in EMT6 tumor-bearing mice (**Figures S6B, C, F**). The reverse of the ratio found in losartan-treated group indicated that losartan reprograms TAMs performing polarization towards M1-like TAMs. The 4T1 tumor cryosections from different treatment groups underwent immunofluorescence staining with CD86 and CD206 antibodies, which are predominantly expressed in M1-like TAMs and M2-like TAMs populations, respectively (**Figure 4A**). The results of the experiment were consistent with flow cytometry quantitation.

**Figure 5:**
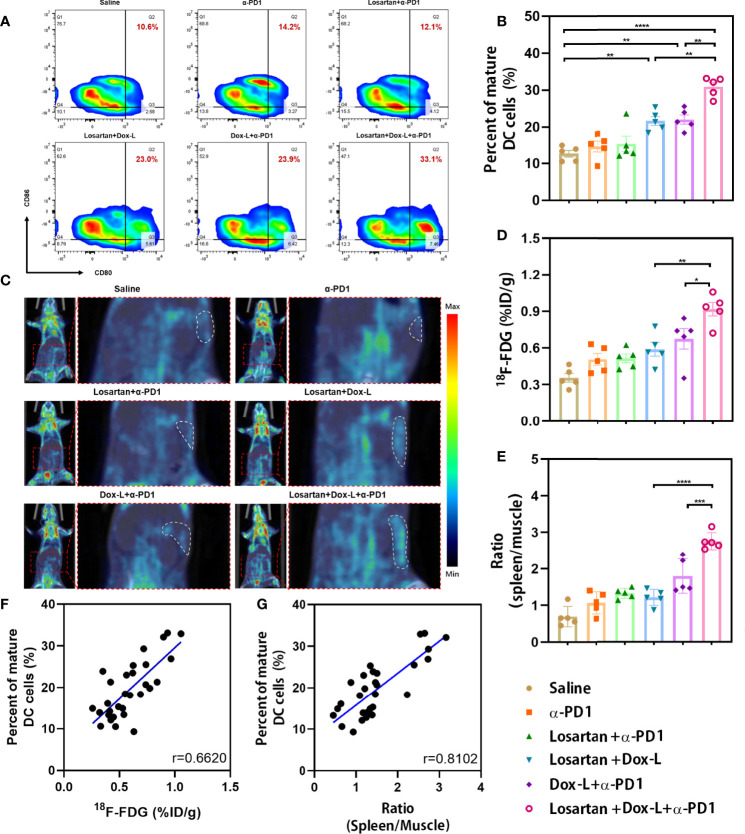
Investigation of the splenic DCs infiltrate and splenic ^18^F-FDG uptake in mice bearing 4T1 tumors after combination therapy. **(A, B)** The percentage of mature DCs (CD11c+, CD80+, CD86+) was analyzed by flow cytometry (n = 5). **(C)** Representative PET/CT images showing the ^18^F-FDG uptake in the spleens of 4T1 tumor-bearing mice at PET scans (n = 5). White dashed line = spleen. **(D)** Splenic ^18^F-FDG uptake was determined by scanning in 4T1 tumor-bearing mice. **(E)** The ratio (spleen/muscle) is expressed as decay-corrected mean SUV of ROI of spleen divided by decay-corrected mean SUV of ROI of muscle. **(F, G)** Correlation of the ^18^F-FDG uptake and the ratio (spleen/muscle) respectively with the number of splenic DC cells present. Data are expressed as the mean ± SEM. Statistical significances were calculated *via* one-way ANOVA using the Tukey post-test, *p < 0.05, **p < 0.01, ***p < 0.001 and ****p < 0.0001.

The effect of the drug combination on ICD marker—CRT expression was observed by immunofluorescence staining. The fluorescence intensity of CRT exposure in 4T1 tumors was significantly enhanced in treatment groups exposed to Dox-L compared with the other groups (**Figures 4A, H**). The ICD effect facilitates DCs maturation, the most crucial antigen-presenting cells, to activate the immune response. Therefore, the spleens of tumor-bearing mice after different therapies were collected and measured by flow cytometry to evaluate the variation of mature DCs (CD11c+, CD80+, CD86+). In 4T1 tumors, the Losartan + Dox-L group and Dox-L + α-PD1 group induced 21.56 ± 1.21% and 21.94 ± 1.26% of splenic DC maturation. In contrast, the combination of Losartan + Dox-L+ α-PD1 improved the mature DC maturation to 30.86 ± 1.20% (*p* = 0.0012 and *p* = 0.0018, respectively), suggesting the combinational strategy efficiently amplified the immune response of chemotherapy and ICB therapy (**Figures 5A, B**). The experiment data of EMT6 models (**Figures S6D, G**) were in accord with 4T1 results. Additionally, this finding is attributed to the presence of Dox-L inducing the ICD effect because experimental Dox-L-treated groups promote DC maturation without exception.

The combination treatment-induced systemic immune response was evaluated by measuring the secretion of serum proinflammatory cytokines with ELISA assay, including tumor necrosis factor-alpha (TNF-α), interferon-gamma (IFN-γ) and interleukin-6 (IL-6), that play vital roles in anti-cancer immunity. The three serum cytokines were significantly increased for 4T1 tumor-bearing mice by treatment with losartan combined chemo-immunotherapy (*p* = 0.0201, *p* = 0.0006 and *p* = 0.0049, respectively), illustrating the establishment of powerful systemic antitumor immune responses (**Figures 4E, I–K**).

To study the immune-memory effect, the mice cured by the Losartan + Dox-L + α-PD1 therapeutic strategy were rechallenged by 4T1 cells on the contralateral mammary fat pad 1 month after the previous tumors disappeared. We found that the mice previously cured by combinational treatment were still resistant to tumor rechallenge, while the tumors of control mice developed rapidly after inoculation (**Figure S7**), demonstrating long-term immunogenic memory function triggered by the combination therapy.

### Splenic ^18^F-FDG PET Imaging Confirms DC Activation

Splenic ^18^F-FDG PET imaging was employed to evaluate the immune response against tumors after different treatments in both 4T1 (**Figure 5C**) and EMT6 (**Figure S8A**) cancer models. ^18^F-FDG PET imaging revealed a significantly increased splenic ^18^F-FDG uptake in Losartan + Dox-L + α-PD1 combination strategy-treated 4T1 tumor-bearing mice when compared to that of Losartan + Dox-L group (*p* = 0.0038) and Dox-L + α-PD1 group (*p* = 0.0472, **Figure 5D**). Also, the spleen-to-muscle ratio calculated by the ^18^F-FDG PET signal in the triple therapy group was 2.76 ± 0.11, significantly higher than that of the Losartan + Dox-L group (1.21 ± 0.10, *p* < 0.0001) and the Dox-L + α-PD1 group (1.80 ± 0.21, *p* = 0.0003, **Figure 5E**). The findings on ^18^F-FDG PET parameters in the 4T1 cancer models can also be extended to EMT6 models (**Figures S8B, C**).

To confirm splenic ^18^F-FDG PET scanning could reflect systemic immune response, we explored the correlation between splenic DC flow cytometry data and the ^18^F-FDG PET parameters of the six treatment groups. The correlation coefficients of splenic ^18^F-FDG uptake with the percentage of mature DCs were 0.6620 in 4T1 tumor-bearing mice (*p* < 0.0001, **Figure 5F**) and 0.4782 in EMT6 tumor-bearing mice (*p* = 0.0075, **Figure S8D**). Furthermore, the spleen-to-muscle ratio demonstrated a significant correlation to the percentage of CD11c+, CD80+, CD86+ DCs in both 4T1 (r = 0.8102, *p* < 0.0001, **Figure 5G**) and EMT6 (r = 0.7489, *p* < 0.0001, **Figure S8E**) mice. Our findings indicated that an effective systemic immune response in tumor models undergoing losartan combined chemo-immunotherapy could be assessed by splenic ^18^F-FDG PET.

## Discussion

**Figure 6:**
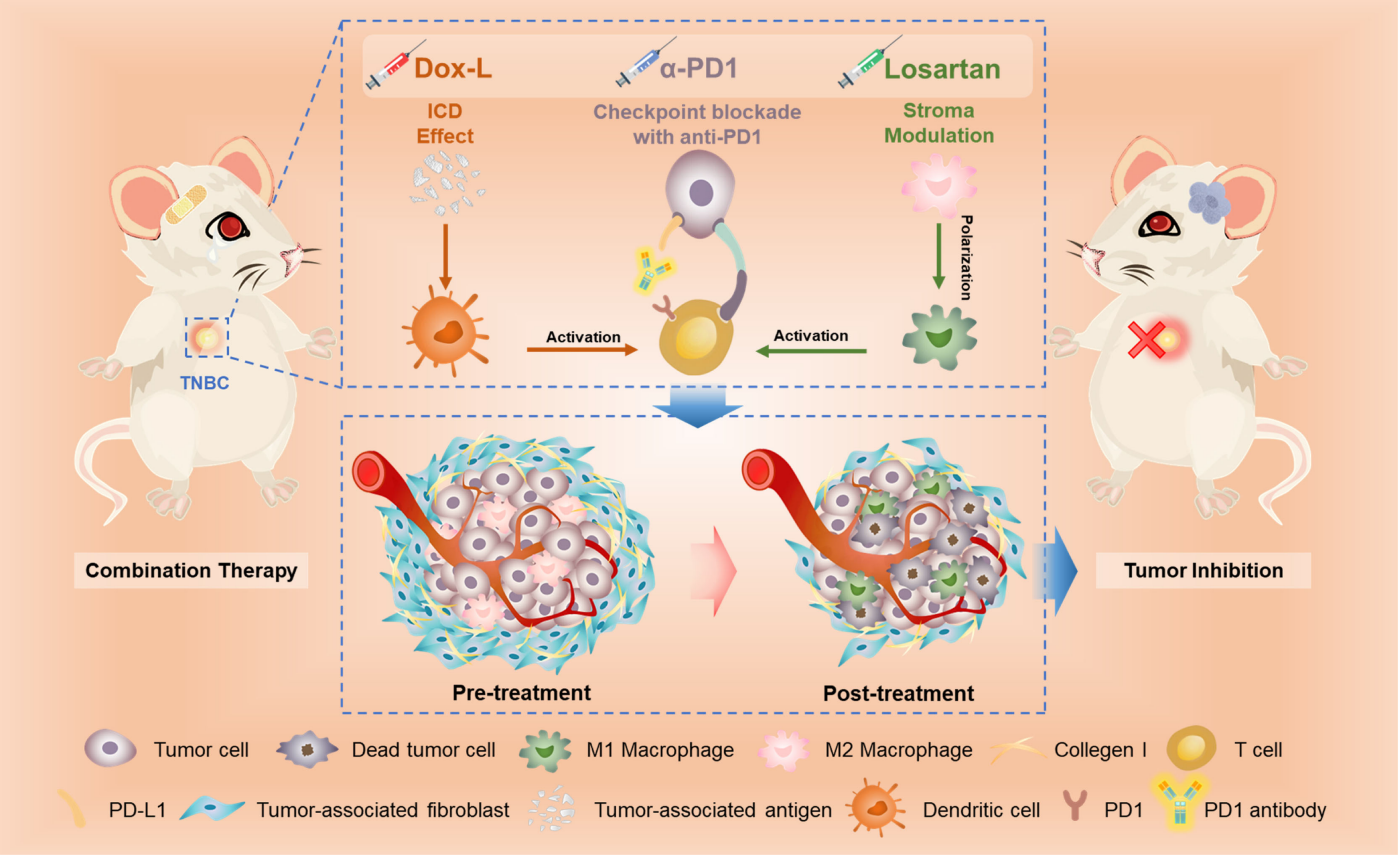
The scheme illustrates the mechanism of losartan-based chemo-immunotherapy to achieve systemic antitumor immune responses.

Our study reported for the first time the effect of tumor inhibition and immune response against TNBC caused by the combined therapeutic strategy (Losartan + Dox-L + α-PD1) to the best of our current knowledge. The study mainly unfolds the coefficient mechanism of antitumor immune activation from losartan, Dox-L and α-PD1 (**Figure 6**).

Given the limited efficacy of PD1 inhibitor monotherapy, considerable efforts have been devoted to improving efficacy with combination therapy to overcome the non-immunogenic nature of TNBC, facilitating immunological function (7, 10). As a rational combination partner, Dox-L triggers the cancer-immunity cycle *via* ICD effect to promote DC maturation to stimulate effector T cell (33, 34), which has been verified in combination with ICB treatment (1, 35, 36). Damage-associated molecular patterns (DAMPs) such as CRT and HMGB1 induced by ICD enable the host immune activation against tumor cells. As a distinct biomarker of ICD, CRT acts as an “eat me” signal, once exposed on the tumor cell surface, to enhance phagocytosis of dying tumor cells and debris (34, 37). We found that Dox-L had the potential to induce significantly higher CRT exposure and HMGB1 excretion in TNBC cells. However, previous literatures demonstrated that Dox-L still lacked the ability to penetrate sufficiently in solid *in-vivo* tumor (21, 22), as evidenced by our result.

It is noted that regulating the aberrant ECM is capable of augmenting drug delivery and reversing immunosuppressive nature. Losartan is a kind of angiotensin II type 1 receptor blockers (ARBs), which serves initially for hypertension treatment. But in previous studies (21, 31, 32), losartan was applied to potentiate delivery of nanomedicine through degradation of dense ECM. Our paper also confirmed that losartan could effectively lower ECM content (e.g. collagen I, α-SMA) and restore compressed micro vessel density (CD31), which were consistent with Vikash’s findings (31, 38). After continuous administration of losartan, Dox-L was proved to gain increased biodistribution in local tumor tissue, which ultimately lead to significantly effective ICD effect, as exemplified by DC maturation level. The function of losartan on degrading dominant components of dense ECM may be attribute to decreased expression of profibrotic signals TGF-β1, CCN2 and ET-1, downstream of angiotensin-II-receptor-1 inhibition (31).

Furthermore, dense stroma and resulting hypoxia are associated with the immune-suppressive tumor microenvironment (26, 39), with polarization of M2 TAMs and insufficient accumulation of cytotoxic immune cells. Several studies pointed out that strategy focusing on modulation ECM and hypoxia status might yield promising results in enhancing anti-tumor immunity (16, 22, 40). The depleting ECM resulted in relieving tumor hypoxia, as verified by immunofluorescence imaging. Here we hypothesize that losartan in combination with chemo-immunotherapy might further enhance immune response. In this study, the results demonstrated that the M2 TAMs related to hypoxia were significantly decreased post-losartan treatment. The data also showed during combined therapy, M1 TAMs cells significantly increased, confirming that tumor immune-nature had reversed from cold to hot. After accounting for PD1 inhibitor, the combinational therapy further added to infiltration and activation of CD8+ T cell. Notably, the combined strategy not only efficiently boosted the antitumor immune responses but also showed long-term immune memory effects in the cured mice, therefore leading to substantial therapeutic efficacy for relapse prevention of the primary tumor.

In previous studies, the researchers cited a similar biological strategy to synthesize new nanomedicines with losartan and chemotherapeutics to achieve better immunotherapy effects (38, 41). Research and development of new nanomedicine brings new breakthrough in cancer treatment. However, compared with mature clinical drug combination strategies, there are still some problems to be solved in the clinical transformation of novel nanoparticles, such as drug biosafety, drug stability, etc. The clinical drug combination method proposed by us is easier to apply in clinical practice, has better biological safety, and is more economical. Clinical biosafety is a challenging concern for drug trial. The agents involved in our strategy are all commonly used in clinic. Indeed, our paper and other researches (21, 31, 32) supported that losartan alone, when used at low doses, did not cause significant tumor cell death neither *in vitro* nor *in vivo*. Moreover, losartan did not induce body weight loss and failed to do harm to major organs in this paper. Losartan was selected over other angiotensin inhibitors in a recently initiated clinical trial involving pancreatic cancer (NCT01821729) at Massachusetts General Hospital (42). The biosafety and low cost of losartan, along with their potentiation of conventional nano-chemotherapy and immunotherapy, makes a perfect candidate for readily approval in clinical patients with indication.

Growing evidence have shown that successful ICB therapy requires a systemic response against cancer involving primary (e.g. the bone marrow) and secondary lymphoid organs (e.g. the spleen) (43–45). However, there is no reliable prediction model available for supervising anti-tumor immune response. Derived from invasive procedures, PD-L1 expression in histological specimen is often deemed as valuable biomarkers for assessment with limited clinical use (46). As a representative “transpathological” approach, molecular imaging with PET achieves the safe and comprehensive evaluation of disease biological processes (47, 48). The systemic immune response was relied on glycolysis cycles to a large extent, therefore the ^18^F-FDG PET has been verified to be potential to monitor immunotherapy-associated metabolic changes (43, 44). However, glucose metabolism is a non-immune cell-specific process. In order to analyze the systemic immune environment more accurately, new specific probes need to be developed. DC activation in the spleen were heavily dependent on abundant glucose and extensive glycolysis to intensify the immunologic response (49, 50). Accordingly, the massive glucose consumption caused by DC maturation makes room for ^18^F-FDG PET detection. In this study, flow cytometry revealed ^18^F-FDG PET parameters, such as ^18^F-FDG uptake and spleen-to-muscle ratio, were correlated strongly with DC maturation status measured by flow cytometry in both 4T1 and EMT6 tumor models. But besides splenic DC transformation, the elevated infiltration of splenic neutrophils and T cells could also lead to increased glucose metabolism, which may be observed with ^18^F-FDG PET imaging (43). Therefore, the topic focusing on the relationship among specific metabolism of various splenic immune cell, splenic PET imaging evaluation and the strength of systemic immune response was needed to be further discussed.

## Conclusion

This study indicated that losartan combined chemo-immunotherapy approach could enhance the antitumor immune-activated efficacy for TNBC by degrading stromal structures, remodeled the immunosuppressive microenvironment, thus resulting in a better therapeutic response. Our findings provide the basis for the translational therapeutic approach for TNBC.

## Data Availability Statement

The raw data supporting the conclusions of this article will be made available by the authors, without undue reservation.

## Ethics Statement 

The animal study was reviewed and approved by the Laboratory Animal Center of Zhejiang University (NO. ZJU20210070 and NO. ZJU20210232).

## Author Contributions

Conception and design, MT, HZ, XH, SL. Development of methodology: QZ,XH, XQ. Acquisition of data (provided animals, provided facilities, etc.), HZ, MT, XH, JW, QZ, XQ and YL. Analysis and interpretation of data (e.g., statistical analysis, biostatistics, computational analysis), QZ, XH, XQ and YL. Writing, review, and/or revision of the manuscript, QZ, XH, MT, HZ, SL, HJ, SW, and RZ. Administrative, technical, or material support (i.e., reporting or organizing data, constructing databases), HZ, MT, XH and JW. Study supervision: HZ, MT and XH. All authors contributed to the article and approved the submitted version.

## Funding

This work was supported by the National Natural Science Foundation of China (81761148029, 81725009, 82030049, 32027802), National Key R&D Program of China (2021YFA110004500, 2021YFE0108300) and Fundamental Research Funds for the Central Universities (2021FZZX002–05).

## Conflict of Interest

The authors declare that the research was conducted in the absence of any commercial or financial relationships that could be construed as a potential conflict of interest.

## Publisher’s Note

All claims expressed in this article are solely those of the authors and do not necessarily represent those of their affiliated organizations, or those of the publisher, the editors and the reviewers. Any product that may be evaluated in this article, or claim that may be made by its manufacturer, is not guaranteed or endorsed by the publisher.
