# Supplementary material for: Enhanced Therapeutic Efficacy of Combining Losartan and Chemo-Immunotherapy for Triple Negative Breast Cancer
Source: Front Immunol. 2022 Jun 23;13:938439. doi: 10.3389/fimmu.2022.938439 (PMC9259940; doi:10.3389/fimmu.2022.938439)
Supplement: Supplementary file 1 [file DataSheet_1.docx]

Supplementary Material

## Supplementary Figures
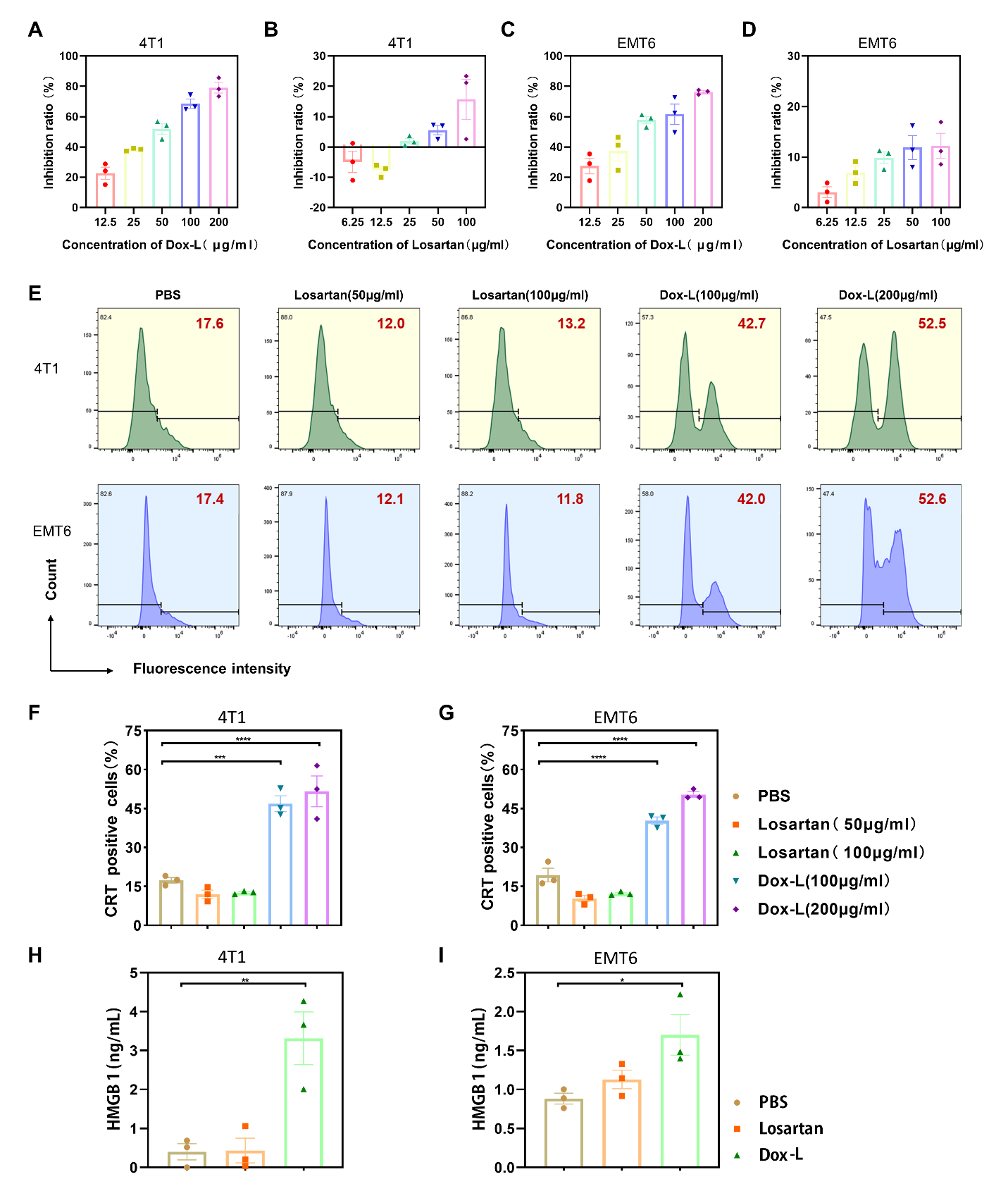


**Supplementary Figure 1.** In vitro analysis of 4T1 and EMT6 tumor models. (A to D) The cell cytotoxicity of 4T1 or EMT6 cells after co-incubation with Dox-L and Losartan for 48h at varying concentrations. (E)Representative surface expression of CRT on (F) 4T1 and (G) EMT6 cells after treatment with PBS, Losartan(50μg/ml), Losartan(100μg/ml), Dox-L(100μg/ml) and Dox-L(200μg/ml) respectively. In vitro measurement of HMGB-1 production by PBS, Losartan and Dox-L treatment in (H) 4T1 and (I) EMT6 cells respectively. Cellular supernatants were used for measurement of HMGB1 levels by an HMGB-1 ELISA kit in the same experiments. Data are expressed as the mean ± SEM. Statistical significances on CRT positive cells were calculated via one-way ANOVA. Statistical significances on HMGB1 release was calculated via Student’s t test, **p* ＜ 0.05, ***p* ＜ 0.01, ****p* ＜ 0.001 and *****p* ＜ 0.0001 (n = 5).


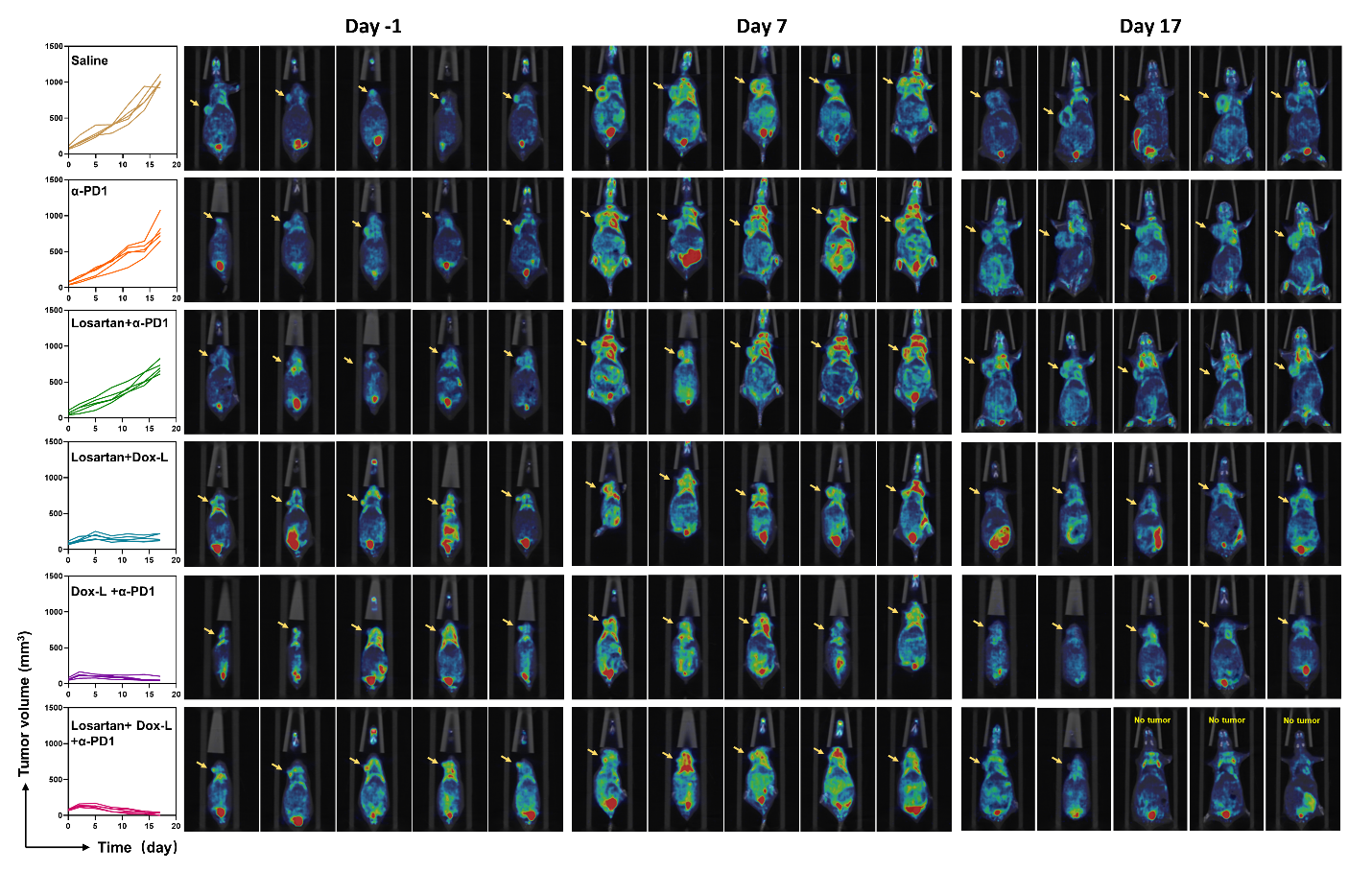


**Supplementary Figure 2.** Individual tumor growth curves of 4T1 tumor bearing mice from different treatment groups of mice and PET/CT imaging of 4T1 tumors at desired time after different treatments (n = 5). The PET/CT images of 4T1 tumor bearing BALB/c mice were taken at Day -1, Day 7 and Day 17, respectively. The yellow arrows indicate the location of tumors.


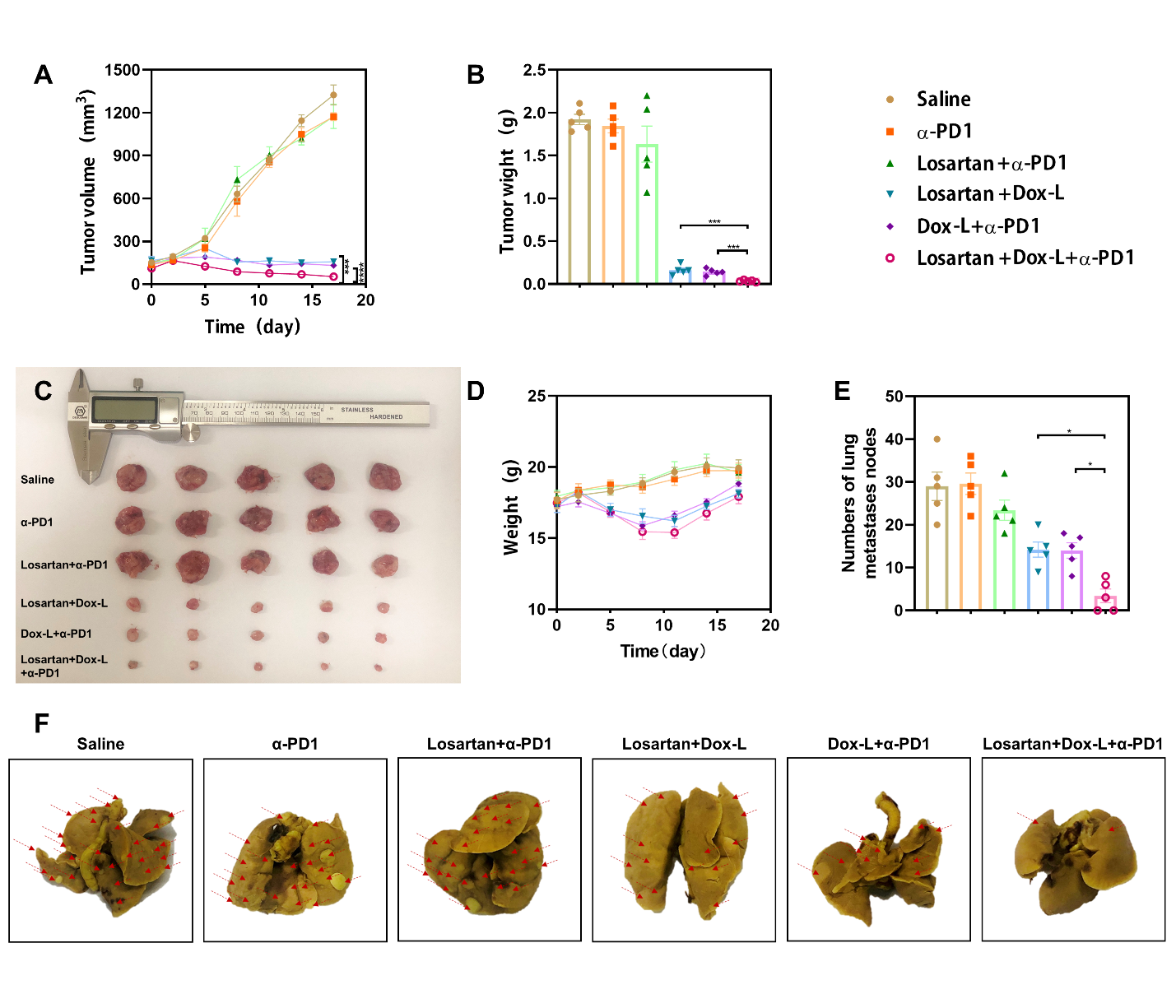


**Supplementary Figure 3.** Anti-tumor effect of losartan, Dox-L plus anti-PD1 immunotherapy in orthotopic EMT6 tumor models. (A) Tumor growth curves of different groups of orthotopic tumor-bearing mice after various treatments as indicated in the figure. (B) Average weights of tumors at the end of treatments. (C) Photographs of excised tumors at the end of treatments. (D) Weight of mice after different treatments. (E) The numbers of lung nodules were counted under anatomy microscope. (F) Representative Lung photographs of EMT6 murine breast tumors treated as indicated. Red arrows indicate the metastatic nodules on the lungs. Data are expressed as the mean ± SEM. Statistical significances were calculated via Student’s t test, **p* ＜ 0.05, ****p* ＜ 0.001 and *****p* ＜ 0.0001 (n = 5).


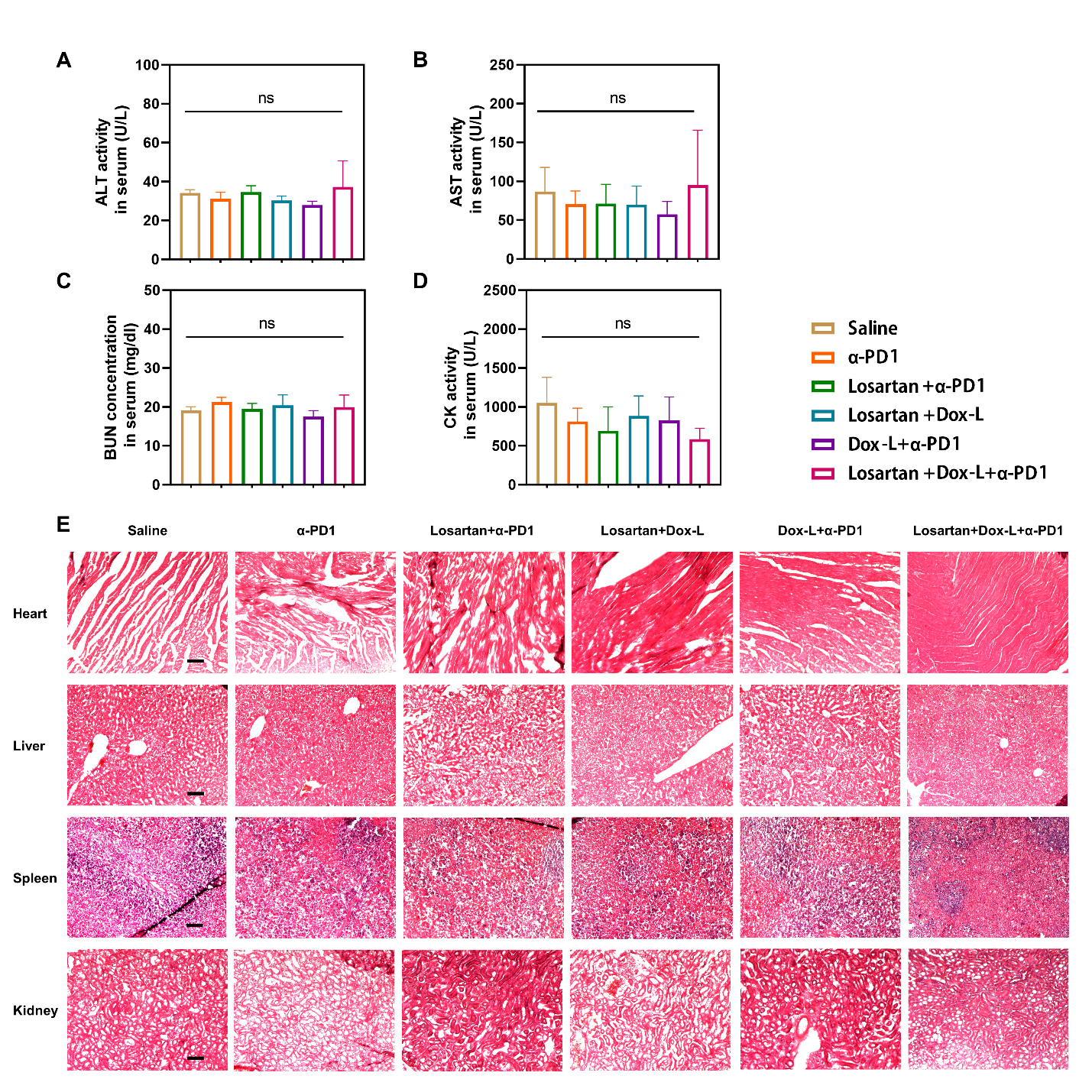


**Supplementary Figure 4.** In vivo biosafety evaluation of the combinational strategy. AST (A), ALT(B), BUN (C) and CK (D) activity in serum (n = 3). (E) H&E-stained tissue sections of major organs (heart, liver, spleen, and kidney) from mice with different antitumor treatments. Scale bar = 100μm.


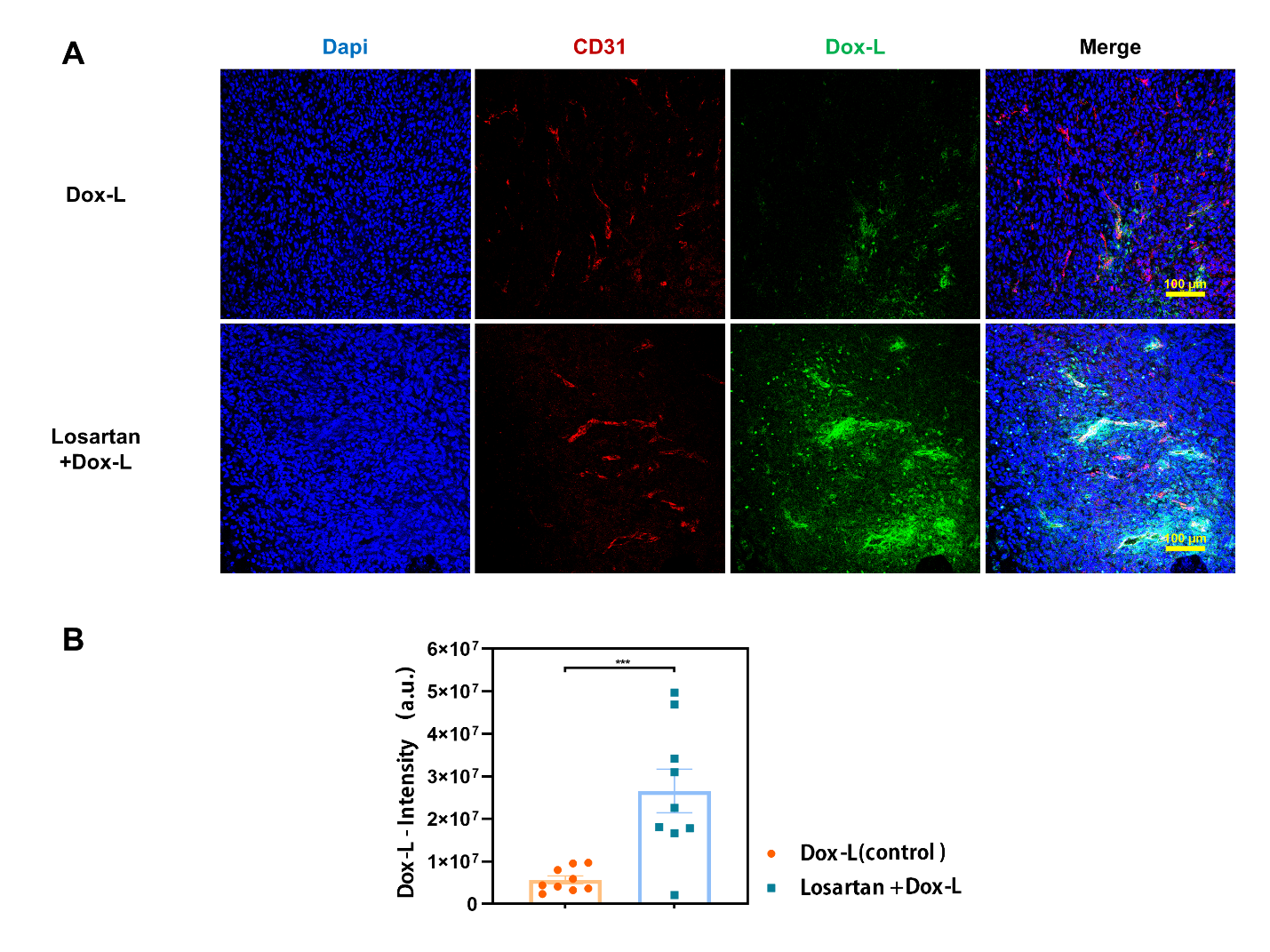


**Supplementary Figure 5.** Situation of drug delivery by the Losartan plus Dox-L compared to Dox-L (control) in an orthotopic 4T1 tumor model. (A) Representative immunofluorescence images of nuclear (blue), CD31 (red) and Dox-L (green) fluorescence at the 4T1 orthotopic tumor site (n = 3) after different treatments as indicated. The mice were sacrificed after 12h for immunofluorescence straining. (B) Quantification of Dox-L fluorescence signals. Scale bar = 100μm. Data are expressed as the mean ± SEM. Statistical significance was calculated via Student’s t test, ****p* ＜ 0.001.


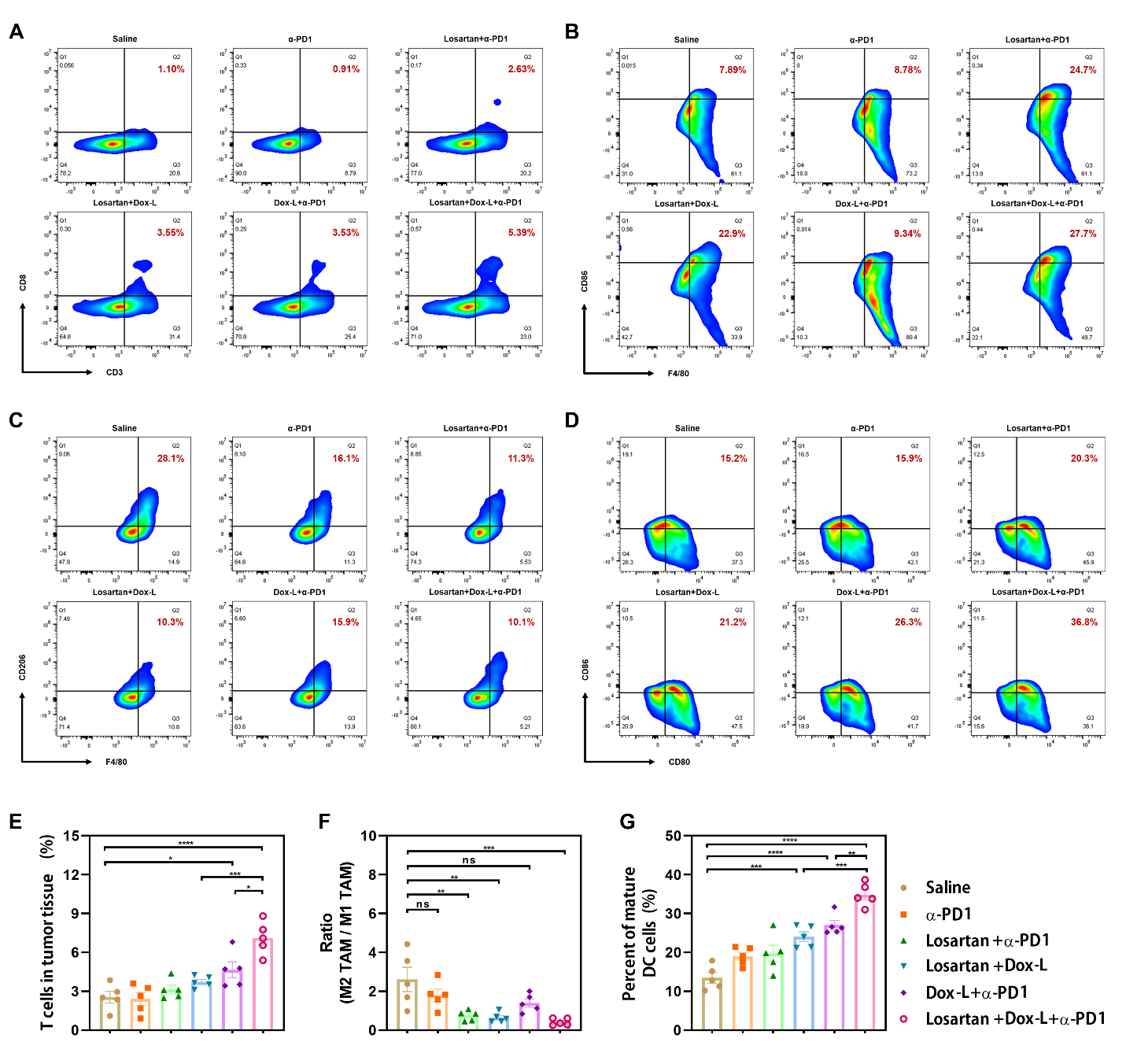


**Supplementary Figure 6.** Losartan combined with chemo-immunotherapy promotes immune-stimulation in orthotopic EMT6 breast tumors. (A to D) Representative flow cytometry plots showing the tumor immune cells, including CD8+ T cells (CD45+, CD3+, CD8+), M1-like TAMs (CD11b+, F4/80+, CD86+), M2-like TAMs (CD11b+, F4/80+, CD206+) in tumors and DCs (CD11c+, CD80+, CD86+) in spleen after different treatments. (E) Quantification of the level of CD8_+_ T cells by flow cytometry analysis (n = 5). (F) Ratio of M2-like TAMs to M1-like TAMs by flow cytometry analysis (n = 5). (G) The percentage of mature DCs (CD11c+, CD80+, CD86+) was analyzed by flow cytometry (n = 5). Data are expressed as the mean ± SEM. Statistical significances were calculated via one-way ANOVA, **p* ＜ 0.05, ***p* ＜ 0.01, ****p* ＜ 0.001 and *****p* ＜ 0.0001.


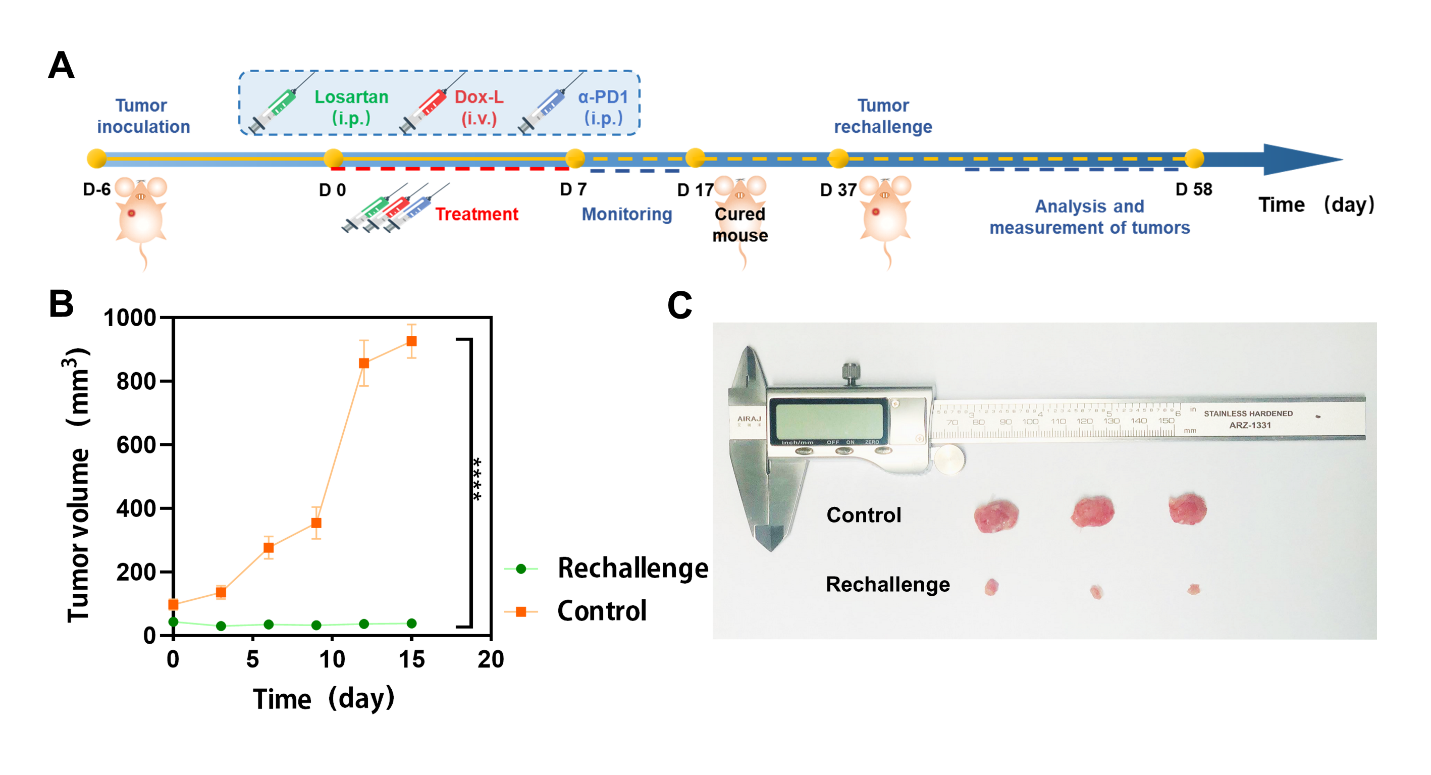


**Supplementary Figure 7.** The immune-memory effect triggered by the combination therapy. (A) Schematic illustration of 4T1 tumor rechallenge for cured mice. (B) Average tumor growth curves of 4T1-tumor-bearing mice from control and rechallenge group (n = 3). (C) Photographs of excised tumors. Tumor volume was measured every 3 days until time to reach a tumor burden of 1000 mm^3^. Data are expressed as the mean ± SEM. Statistical significance was calculated via Student’s t test, *****p* ＜ 0.0001.


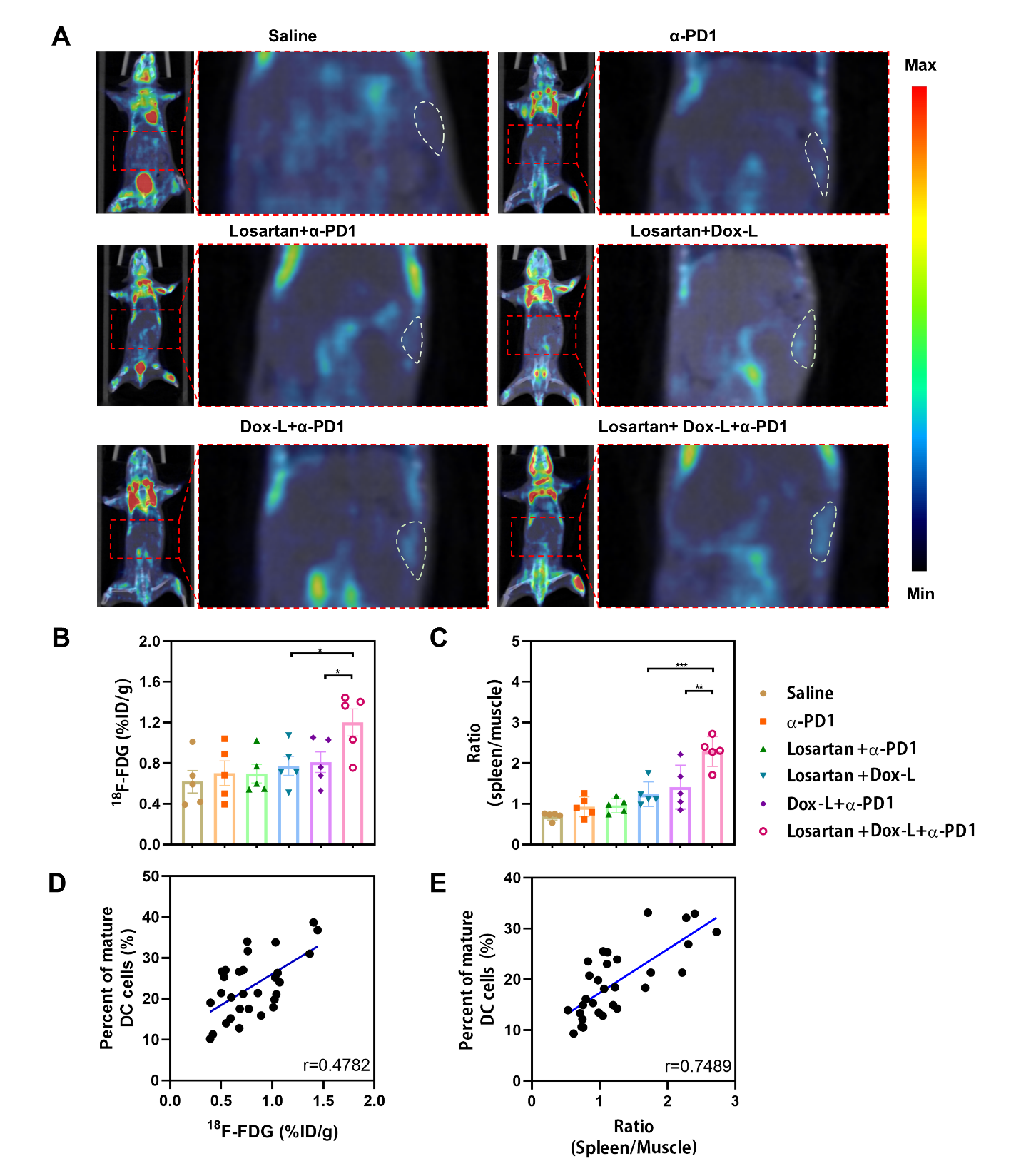


**Supplementary Figure 8.** The immune-memory effect triggered by the combination therapy. (A) Schematic illustration of 4T1 tumor rechallenge for cured mice. (B) Average tumor growth curves of 4T1-tumor-bearing mice from control and rechallenge group (n = 3). (C) Photographs of excised tumors. Tumor volume was measured every 3 days until time to reach a tumor burden of 1000 mm^3^. Data are expressed as the mean ± SEM. Statistical significance was calculated via Student’s t test, *****p* ＜ 0.0001.
